# Auto-Intoxication of Intestinal Origin*Read before the Louisville Medico-Chirurgical Society, October 21, 1898.

**Published:** 1898-12

**Authors:** Thomas Hunt Stucky

**Affiliations:** Professor Principles and Practice of Medicine in the Hospital College of Medicine, etc., Louisville, Ky.


### AUTO-INTOXICATION OF INTESTINAL ORIGIN.*

BY THOMAS HUNT STUCKY, M.D.

Professor Principles and Practice of Medicine in the Hospital College of Medicine, etc., Louisville, Ky.

We are taught by Bouchard that the poisons contained in the intestines, and also those which come from foods, bile or putrefaction, enter into the complex intoxication called uremia.

We therefore infer that if the quantity of poison increases in the intestines, an intoxication becomes possible, at the same time that disassimilation does not hand over to the blood a larger amount of toxic material, even when the kidney remains permeable.

Investigation by Bouchard, Senator, and others has proven to us that reabsorption of substances contained in the digestive canal, without the presence of other pathological states, produces a toxicity or toxemia mixed in character and dependent upon one of the following sources:

1. Foods, even the most inoffensive in character and appearance, and the flesh of muscles are toxic, principally due to the mineral matters and potassium they contain.

2. Bile contains poison. The eight hundred or one thousand grammes of bile which are turned each day into the intestines of an adult of average weight are toxic on account of their coloring matter principally, *e. g.*, bilirubin and also other substances, some known, such as the biliary salts, others unknown.

3. Putrefactions which develop in the alimentary residues produce poison.

The injection of 2.5 grammes of putrefied meat is sufficient to kill.

4. Fecal matter is toxic; this toxicity is due chiefly to potass. and ammonia; this represents about one-fifth of the total toxicity to the union of organic principles, in which are included alkaloidal substances.

We know, then, from the preceding statement of Bouchard, demonstrated quite conclusively, that in normal or physiological conditions there is material for intoxicating, and how with a kidney functionally free, if the production of toxic material is accidentally abundant, it may accumulate in the blood in the proportion capable of causing symptoms of intoxication to arise. When fermentation has become active in the whole length of the digestive tube, we see produced characteristic phenomena.

The unusual development of gas determines abdominal meteorism and tympanites, arising either from stomach or intestines, or may be carried to the latter from the former.

---

*Read before the Louisville Medico-Chirurgical Society, October 21, 1898.

May have eructations, belching, preceded by burning sensations in the stomach, or may have pyrosis in the esophagus or pharynx.

May have acid vomiting, the acidity of which is due to acetic acid, rarely hydrochloric. Changes in the teeth may be due to mouth acidity.

Contents of intestines may have become abnormally acid, provoking diarrhea by irritation of mucus membrane, but also irritative to the skin outside of the rectum, as in the acid dyspepsia of infants.

The red tongue being another prominent evidence.

Here there is substituted an acid reaction of the intestinal contents for the normal.

There is change in color of the stools; bile is expelled of a green color; sulphureted hydrogen is diminished.

Bismuth given with the idea of diminishing the diarrhea, gives no longer to the dejecta a black color, for there is no longer formed sulphide of bismuth.

There are ocular demonstrations of the production of acid fermentation in the digestive canal.

When fermentation of putrid character predominates, an excessive disengagement of sulphureted hydrogen, ammonia and its sulphate, which manifest themselves to our olfactories.

Parallel to these objective phenomena, there exist those of a subjective character, viz.: fatigue, headache, buzzing in the ears and deafness, disturbances of sight, vertigo, and depression.

With a healthy kidney acting well there may be no further trouble, but if the renal elimination is insufficient, may have evidences of uremic intoxication, through simple exaggeration of intestinal fermentation.

If, as illustrated by Bouchard, abundant vomiting has produced oliguria, we may have coldness established, paralysis of the vessels of the skin, cramps, convulsions, coma, paralysis, death even, while the kidney itself may not be really diseased.

For the development of such accidents, it is only necessary that the quantity of toxic material introduced into the blood should exceed the eliminating power of the kidney.

The variations of urinary toxicity may be in similar cases the measure of the degree of toxicity, depending on the amount of urine eliminated; and we find under intestinal fermentation an increase in the toxicity of the urine.

If intestinal fermentation is controlled or suppressed, there is a lessening of urinary toxicity.

There is only a lessening, not a disappearance, because only one of the natural sources of the toxicity is controlled.

The toxicity of the urine can be lessened by neutralizing the products of putrefaction, by acid or charcoal, preventing their absorption, or by preventing putrefaction through intestinal antisepsis by means of beta-naphthol, salol, benzosol, or iodoform.

This fact has also been proven by chemistry, by Staedler (1848) finding phenol in the urine; 1877, Bauman found phenol in fecal matter, this no doubt passing from the digestive tube into the urine.

1826, Tildeman and Greulin, in the duodenum, discovered a substance which gave a red color with chlorinated water, indol.

Senator has more recently confirmed the above in his analysis of meconium. He does not find indican in the urine or idol in meconium.

Martin established that in every disease of the intestinal tract there was increase in urinary indican.

Salgowsky made researches and found phenol and cresol.

We see them increase like indican in the urine in certain forms of diarrhea and in intestinal obstruction.

It has been observed by Senator, Riess and Litten not only in diabetes mellitus, leucocythemia, but in grave dyspeptic states, carcinoma of stomach, all cases in which anomalous fermentation is produced in the digestive canal.

We know, therefore, that if these putrid substances are found in excess, there may result an intoxication without disease of the kidney.

It is natural, then, to ask if there is no other protection than the kidney—possibly the liver.

The experiments of G. H Roger in his injections of extract of putrid meat into the portal vein, found less toxic influence than when injected into the general circulation.

We can but conclude that the liver is an organ of protection to the economy; that it arrests more or less the general toxic effect.

Other channels may be by rapid intestinal action, active diarrhea, or the forming of the intestinal contents into a hard fecal bolus, almost inoffensive because it no longer allows for absorption.

The breath and skin of the patient have a disagreeable odor. Where the toxemia is of chronic type, there is an ashen hue due to the toxic pigment contained in the blood.

Bouchard explains the fever following an aseptic laparotomy as due to toxemia, and points to the relief usually obtained by intestinal evacuation.

Special forms of intoxication have been observed following the formations of sulphureted hydrogen in excess; the ingestion of fish, preserved goose, and sausage.

A principle analogous to atropine, producing redness, mydriasis, and dryness of the skin, was developed from the sausages.

Various psychic disturbances, chlorosis, and inanition are among the sequelæ of auto-toxemia.

The prognosis necessarily depends upon the cause. Where the retained material can be eliminated and the absorption cut short, the system is given a chance to react from the primary trouble.

Thus lavage in gastric dilatation does not cure the non-retracting stomach, but it relieves the headache, restlessness, etc., and permits assimilation.

Constipation represents the mildest form and strangulation the severest form of intoxication.

Absorption necessarily ceases when the feces become hardened, as in constipation.

The treatment of auto-intoxication is almost specific.

Since we are treating a symptom and not a disease, we must expect relief and not a cure always.

Prophylaxis consists not only in the avoidance of high temperature and the ingestion of pure foods, but also in the eschewing of such foods as each individual has found to be indigestible. Thus potatoes may be perfectly digestible by one person, yet another, apparently normal person, may fill with gas after their ingestion.

Lavage and enemata are the speediest ways of removing the poisonous material from the alimentary canal.

Emetics are dangerous, since they may be followed by oliguria, permitting the poison already absorbed to remain in the system. Non-depressing salines are useful.

The antipyretic becomes a matter of individual choice.

Among the favorites may be mentioned beta-naphthol, charcoal, salol—arsenite of copper—thymol, guaiacol and its compounds, boric acid, and the mineral acids.

Lavage, enemata, and auto-zymotic, and stimulation seem to formulate the approved treatment.—*American Practitioner and News.*

